# Prevalence, Risk Factors, and Result Features in the Detection of Latent Tuberculosis Infection in Thai Healthcare Workers Using QuantiFERON-TB Gold Plus

**DOI:** 10.7759/cureus.60960

**Published:** 2024-05-23

**Authors:** Wiphat Klayut, Sopa Srisungngam, Sirilada Suphankong, Pantip Sirichote, Benjawan Phetsuksiri, Supranee Bunchoo, Chiranan Jakreng, Savitree Racksas, Ballang Uppapong, Janisara Rudeeaneksin

**Affiliations:** 1 Department of Medical Sciences, Ministry of Public Health, National Institute of Health, Nonthaburi, THA; 2 Office of the Permanent Secretary, Somdej Phra Phutthaloetla Hospital, Ministry of Public Health, Samut Songkhram, THA

**Keywords:** healthcare worker, infection, risk factors, prevalence, quantiferon-tb gold plus, latent tuberculosis

## Abstract

Introduction

Latent tuberculosis infection (LTBI) is an enormous reservoir for tuberculosis (TB), and healthcare workers (HCWs) are at high risk for TB infection. QuantiFERON-TB Gold Plus (QFT-Plus) is an alternative to the tuberculin skin test for LTBI detection, but data on its application and LTBI detected by QFT-Plus in high TB burden countries are limited. This study aimed to determine the prevalence of LTBI and its risk factors, and to investigate the QFT-Plus results in Thai HCWs.

Methods

A cross-sectional analytical study was conducted among HCWs at a secondary care hospital in Health Region 5, Thailand. Eligible HCWs were enrolled and underwent QFT-Plus testing. Interferon-gamma (IFN-γ) values in tubes were analysed. The prevalence and associated risk factors for LTBI were assessed based on laboratory and sociodemographic data. Logistic regression analyses were applied to calculate odds ratios (OR, aOR) reported with 95% confidence intervals (CI).

Results

Of the 269 participants enrolled, their median age was 42 years and 93.31% (n = 251/269) were female. The majority (n = 178/269, 66.17%) were nurses or nurse assistants and 42.75% (n = 115/269) worked in the inpatient medical wards. Overall, the QFT-Plus results showed 110 (40.89%) positive with good agreement (93.68%; κ 0.87) and high correlation (Spearman’s ρ 0.91) of IFN-γ concentrations in the two antigen tubes. A true difference in IFN-γ values for predicting a recent infection was found about 7.81% (n = 21/269). By univariate and multivariate analyses, the participants’ age > 40 years (OR = 3.21, 95% CI: 1.84-5.64%; aOR = 2.05, 95% CI: 1.07-3.96%), and employment duration > 10 years (OR = 3.19, 95% CI: 1.66-6.37%; aOR = 2.34, 95% CI: 1.05-5.21%) were significantly associated with the increased risk of LTBI (p*-*value < 0.05).

Conclusions

The prevalence of LTBI among these HCWs was high, and the increased risk factors for LTBI according to QFT-Plus positivity were age over 40 years and working time in the hospital for more than 10 years. It is important to screen HCWs in this setting for LTBI, particularly those with long employment durations and older ages. The high prevalence of LTBI suggests that LTBI management, such as regular screening and treatment, should be considered together with strengthening preventive measures, especially in high-risk groups.

## Introduction

Tuberculosis (TB) remains a major public health threat worldwide, with an estimated 10.6 million new TB cases and 1.3 million deaths in 2022 [1]. Approximately a quarter of the world’s population has latent TB infection (LTBI) [2], an asymptomatic state of *Mycobacterium tuberculosis* (MTB) infection that is not infectious. In cases of LTBI, about 5-15% can develop active TB disease during their lives, and this is more likely to occur in the first two years after MTB infection [3]. To control and diminish TB, LTBI screening, effective infection prevention, and the prevention of LTBI progression to active TB disease are essential, especially in at-risk populations.

Accurate diagnosis of LTBI is challenging due to the lack of a gold standard test [4]. The WHO has established the detection of LTBI in high-risk populations using the tuberculin skin test (TST) or interferon-gamma (IFN-γ) release assays (IGRAs) [5]. Both methods can identify LTBI by detecting indirect immunological responses against MTB antigens, assuming that the immune response has developed after MTB infection. The TST, an old skin test that generates a delayed hypersensitivity reaction and produces an induration, was a common method used to diagnose TB infection due to its low cost and ease of use but it has known limitations, such as the requirement for two visits and the cross-reactivity with the previous Bacillus Calmette-Guerin (BCG) vaccination and non-tuberculous mycobacterial infections [6]. In countries where BCG vaccination has high coverage, interference between BCG and TST reactions is commonly found, which impacts the test’s specificity [7]. IGRAs are alternatives to TST that measure blood levels of IFN-γ released by T lymphocytes in response to specific MTB antigens in tubes [6]. Compared to the TST, IGRAs are more costly but have higher specificity for detecting TB infection [8]. IGRAs include T-SPOT.*TB*, an enzyme-linked immunospot test, QuantiFERON-TB Gold in tube (QFT-GIT), and QuantiFERON-Plus (QFT-Plus) [9]. The QFT-Plus test is a new generation of enzyme-linked immunosorbent assay (ELISA)-based IGRAs, introduced to replace its predecessor, QFT-GIT (QIAGEN, Hilden, Germany). This new version contains an additional antigen tube (TB2) for detecting the IFN-γ released from both CD4+ T helper and CD8+ cytotoxic T lymphocytes [9]. The manufacturer claims that QFT-Plus has improved sensitivity due to the stimulation of both CD4+ and CD8+ T lymphocytes. Recently, the evaluation of CD8+ T cell activity, as measured by the difference in TB2-TB1 responses, showed a significant association with recent exposure to TB, suggesting CD8+ T cell response in QFT-Plus as a marker of recent infection [10]. However, the QuantiFERON tests (QFT-GIT, QFT-Plus) have some limitations such as high cost, complexity, and the negative effect caused by immunological factors, with only a slight advantage of QFT-Plus over QFT-GIT in positive rate documented [8]. Despite the higher cost and some challenges, its utility is expected to increase in high TB burden countries [6]. To date, studies on the QFT-Plus utility and LTBI have largely been performed in low and middle-TB incidence countries, while data on its applications, including the positive rate and factors associated with test positivity in high TB burden settings, are limited.

Thailand is a high TB incidence country, with an estimated 111,000 TB cases, or 155 per 100,000 population, in 2022 [1] in a nation of approximately 73 million people. LTBI is a major reservoir for active TB; therefore, accurate detection and appropriate management of LTBI are important for reducing TB incidence [8]. Healthcare workers (HCWs), especially in countries with high TB prevalence, are at increased risk of TB infection and developing the disease due to their frequent, prolonged, and close exposure to infectious TB cases, particularly when performing high-risk medical procedures [11]. The higher risk of TB infection also increases with poor practice or limited infection prevention and control measures, such as inconsistent or no use of N-95 masks and inappropriate use of personal protective equipment (PPE) during direct contact with TB patients to prevent TB acquisition. The incidence of LTBI is estimated to be high among HCWs, showing a two to three times increase over the general population [12]. Therefore, high-risk groups of HCWs should be tested for TB infection, and risk factors for LTBI should be assessed. A systematic review based on data from January 2005 to June 2017 on LTBI in HCWs in low- and middle-income countries reported that factors associated with LTBI were work duration, work location, TB contact, and job category, while a few studies reported on the factor of infection control measures in healthcare facilities [13]. The prevalence and associated factors can be affected by different tests, settings, populations, and times. Data on the prevalence and risk factors for LTBI are valuable as they can be used to design and implement effective interventions for TB infection prevention and control. Concurrently, the COVID-19 pandemic has affected TB services, including TB infection screening and prevention, impeding the progress of the End TB Strategy goals [14]. This study aimed to estimate the LTBI prevalence among Thai HCWs and assess risk factors associated with LTBI and the relationship with CD8+ T cell response according to QFT-Plus testing. Baseline data on LTBI and QFT-Plus result features in Thai HCWs at the time before the COVID-19 pandemic was generated.

## Materials and methods

Subjects and study design

A cross-sectional analytical study was conducted among HCWs at Somdej Phra Phutthaloetla Hospital, a secondary care center in Health Region 5, located in Samut Songkhram Province, adjacent to Bangkok, Thailand. The hospital has been designated as one of the regional secondary or tertiary care facilities among 13 health regions across the country. HCWs who applied for LTBI screening were randomly enrolled. The recruited participants consisted of HCWs engaged in medical professions, including clerk officers and housekeepers in medical care, working at different locations in the hospital for more than 6 months. HCWs with previously diagnosed TB disease were excluded. Sociodemographic data collected included gender, age, job type, department or ward, and years of employment. Prior to LTBI testing, active TB screening was conducted by clinical evaluation and chest radiography as part of a routine annual health check-up program. TB cases were not included in the QFT testing. Blood samples were collected and tested for LTBI using QFT-Plus. Risk factors were determined by logistic regression analyses. Odds ratios (ORs and aORs) were calculated and reported with 95% CIs.

Blood sample collection and QFT-Plus assay

The QFT-Plus assay was manually performed and the results were interpreted according to the manufacturer’s instructions. Briefly, 4 ml of venous blood was collected by venipuncture and then transferred up to the 1 ml mark of each blood collection tube in the following order: Nil (negative control), TB1 (contains antigenic peptides from the MTB complex-associated antigens - early secretory antigenic target (ESAT)-6, and culture filtrate antigen (CFP)-10 - to elicit cell-mediated immune [CMI] responses from CD4+ T helper lymphocytes), TB2 (contains peptide antigens like TB1 plus an additional set of peptides targeted to the induction of CMI responses from CD8+ cytotoxic T lymphocytes), and Mitogen (positive control), and then mixed gently. The samples were transported to the Regional Medical Sciences Center 5 Samut Songkhram (RMSc 5) for immediate incubation at 37 ± 1 °C for 16 to 24 hours. The plasma was then separated by centrifugation at 3,000 x g and stored at 4 °C before shipment for further analysis. The QFT ELISA was conducted at the National Institute of Health, Nonthaburi Province, Thailand. The ELISA results were converted into international units per milliliter (IU/ml) and interpreted using the software supplied by the manufacturer. A QFT-Plus result was defined as positive when IFN-γ concentrations of either the TB antigen (TB1 or TB2) minus Nil tube were ≥ the cut-off value of 0.35 IU/ml and ≥ 25% of the Nil value. All reported IFN-γ concentrations were Nil-corrected. With the upper limit of linearity for the assay being 10.0 IU/ml, IFN-γ concentrations exceeding 10.0 IU/ml were designated as 10.0 IU/ml. The result was considered indeterminate if the antigen-stimulated sample showed negative IFN-γ and the positive control IFN-γ value was < 0.5 IU/ml after subtracting the negative control or the Nil IFN-γ value. Participants with a positive QFT-Plus were considered to have LTBI.

All HCWs were informed about the QFT-Plus results through the laboratory reporting document managed by the Laboratory and Occupational Health Offices. For those with a positive QFT-Plus result, further details regarding the LTBI were provided and medical attention was recommended.

Data and statistical analysis

The frequencies and prevalence of LTBI were assessed. Prevalence, defined as the number of individuals with LTBI divided by the total number of individuals in the study, was reported as a percentage. Stata/IC 14.2 for Windows (StataCorp LLC, College Station, TX, USA) was used for statistical analyses. The agreement between the TB1 and TB2 detection was evaluated by computing the overall percentage of concordant QFT-Plus results and Cohen’s κ coefficient (κ, kappa); below 0.0 is poor, 0.00-0.20 is slight, 0.21-0.40 is fair, 0.41-0.60 is moderate, 0.61-0.80 is substantial, and 0.81-1.00 is almost perfect, with a 95% CI. Spearman’s rank correlation was used to calculate the correlation between IFN-γ concentrations in the TB1 and TB2 tubes. Univariate and multivariate logistic regressions were applied to calculate the crude (unadjusted) OR and aOR, respectively. The univariate analysis identified independent factors or characteristics associated with QFT-Plus positivity or LTBI. In the multivariate analysis, risk factors for LTBI adjusted for potentially confounding variables were further assessed. Characteristics selected for the multivariate logistic regression model had p-values < 0.15 in the univariate analyses. The same analysis was performed for characteristics denoting whether a difference of IFN-γ concentrations between the TB2 and TB1 tubes (TB2 - TB1) was > 0.6 IU/ml, assessing the specific response of CD8+ cytotoxic T lymphocytes. A difference in these IFN-γ concentrations > 0.6 IU/ml was defined as a positive result for the true difference to avoid bias due to the intrinsic variability of the test [9]. The true difference between positive TB1 and TB2 detection also indicated recent TB infection according to the manufacturer’s indications. Microsoft Excel 365 was used to create a scatterplot of the IFN-γ concentrations of TB1 and TB2. For data comparisons, all reported p-values were two-tailed and calculated with statistical significance at p-values < 0.05.

Ethical approval

This study was approved by the ethical committee of Somdej Phra Phutthaloetla Hospital (Reference no. 04/2562). All HCWs consented to participate voluntarily, and identified LTBI cases were recommended for medical attention.

## Results

Characteristics of studied HCWs

A total of 269 eligible HCWs were enrolled. The median age was 42 years (interquartile range [IQR] 37-50), and almost all were female (n = 251, 93.31%). More than half of the participants (n = 155, 57.63%) were aged over 40 years, with those aged 40-49 years (n = 98, 36.43%) being the largest group, followed by those aged above 50 years (n = 70, 26.02%). The youngest and oldest HCWs were 20 and 59 years, respectively. The vast majority of HCWs were nurses or nurse assistants (n = 178, 66.17%). Nearly half worked in inpatient wards (n = 115, 42.75%). Others, at about 10% each, worked as office clerks/housekeepers, patient care personnel, and medical technologists (Table 1). The median employment duration was 20 years (IQR 9-26). Based on working duration, those employed for 11-20 years accounted for 30.48% (n = 82/269), followed by those working for 21-30 years, who accounted for 27.14% (n = 73/269). Most had extensive experience, as the majority (n = 197; 73.23%) had been employed for more than 10 years. The longest working period was 40 years, while the shortest was about 1 year. The sociodemographic characteristics of the studied HCWs are summarized in Table 1.

**Table 1:** Demographic characteristics of HCWs in this study. HCWs: Healthcare workers; n: Number.

Characteristics	n (%)
	269 (100.0)
Gender	
Female	251 (93.31)
Male	18 (6.69)
Age (years)	
20-29	40 (14.87)
30-39	61 (22.68)
40-49	98 (36.43)
≥ 50	70 (26.02)
Employment	
Office clerk/Housekeeper	37 (13.75)
Registered nurse/Nurse assistant	178 (66.17)
Patient care personnel	27 (10.04)
Medical technologist	27 (10.04)
Department	
Inpatient	115 (42.75)
Outpatient	90 (33.46)
Intensive care unit	40 (14.87)
Laboratory	24 (8.92)
Employment duration (years)	
< 1	3 (1.12)
1-10	69 (25.65)
11-20	82 (30.48)
21-30	73 (27.14)
> 30	42 (15.61)

QFT-Plus results and LTBI prevalence in HCWs

QFT-Plus yielded valid results for all 269 HCWs with no indeterminate outcomes. Of these, 110 HCWs were categorized as QFT-Plus positive (40.89%) and considered to have LTBI. Stratified data showed varying LTBI prevalence across the following categories: (1) Gender: Female (n = 101/251; 40.24%) versus Male (n = 9/18; 50.0%); (2) Age (years): ≤ 40 years (n = 29/114, 25.44%) versus ≥ 40 years (n = 81/155, 52.26%); (3) Employment (job): Office clerk/Housekeeper (n = 19/37, 51.35%) versus Registered nurse/Nurse assistant (n = 68/178, 38.2%), Patient care personnel (n = 12/27, 44.44%), or Medical technologist (n = 11/27, 40.74%); (4) Department (working location): Inpatient ward (n = 42/115, 36.52%) versus Outpatient ward (n = 44/90, 48.89%), Intensive care unit (n = 13/40, 32.5%), or Laboratory (n = 11/24, 45.83%); and (5) Employment duration (years): ≤ 10 years (n = 16/72, 22.22%) versus ≥ 10 years (n = 94/197, 47.72%).

The highest prevalence of LTBI was among office clerks/housekeepers (n = 19/37, 51.26%) and those working in outpatient wards (n = 44/90, 48.89%). Those who were over 40 years old (n = 81/155, 52.26%) and had worked more than 10 years (n = 94/197; 47.72%) were significantly more likely to be infected with LTBI (P-value < 0.05). In contrast, the lowest prevalence of LTBI was among those aged under 40 years (n = 29/114, 25.44%) and those with a working duration of less than 10 years (n = 16/72, 22.22%). The distribution of positive QFT-Plus results and the LTBI prevalence among Thai HCWs in this study are summarized in Table 2.​​​​​​​

**Table 2:** Univariate and multivariate logistic regression analyses to identify factors associated with QFT-Plus positivity. QFT-Plus: QuantiFERON-TB Gold Plus; IU/ml: International unit per milliliter; n: Number; OR: Odds ratio; aOR: Adjusted odds ratio. ^a^p-value < 0.05 was considered statistically significant.

Characteristics	Total	QFT-Plus positive (n =110)				
	(n=269)	n (%)	OR (95% CI)	p-value	aOR (95% CI)	p-value
Gender						
Female	251	101 (40.24)	Reference		Reference	
Male	18	9 (50.0)	1.48 (0.50-4.38)	0.42	ND	-
Age (years)						
≤ 40	114	29 (25.44)	Reference		Reference	
> 40	155	81 (52.26)	3.21 (1.84-5.64)	0.00^a^	2.05 (1.07-3.96)	0.03^a^
Employment						
Office clerk/Housekeeper	37	19 (51.35)	Reference		Reference	
Registered nurse/Nurse assistant	178	68 (38.2)	0.59 (0.27-1.27)	0.14	0.65 (0.29-1.42)	0.28
Patient care personnel	27	12 (44.44)	0.76 (0.25-2.3)	0.59	0.89 (0.3-2.59)	0.83
Medical technologist	27	11 (40.74)	0.65 (0.21-1.98)	0.40	0.22 (0.02-2.3)	0.21
Department						
Inpatient	115	42 (36.52)	Reference		Reference	
Outpatient	90	44 (48.89)	1.66 (0.91-3.03)	0.08	1.59 (0.86-2.94)	0.14
Intensive care unit	40	13 (32.5)	0.84 (0.36-1.9)	0.65	0.70 (0.32-1.55)	0.38
Laboratory	24	11 (45.83)	1.47 (0.54-3.91)	0.39	3.71 (0.32-42.91)	0.29
Employment duration (years)						
≤ 10	72	16 (22.22)	Reference		Reference	
> 10	197	94 (47.72)	3.19 (1.66-6.37)	0.00^a^	2.34 (1.05-5.21)	0.04^a^

Result agreement between TB1 and TB2 responses

**Figure 1:**
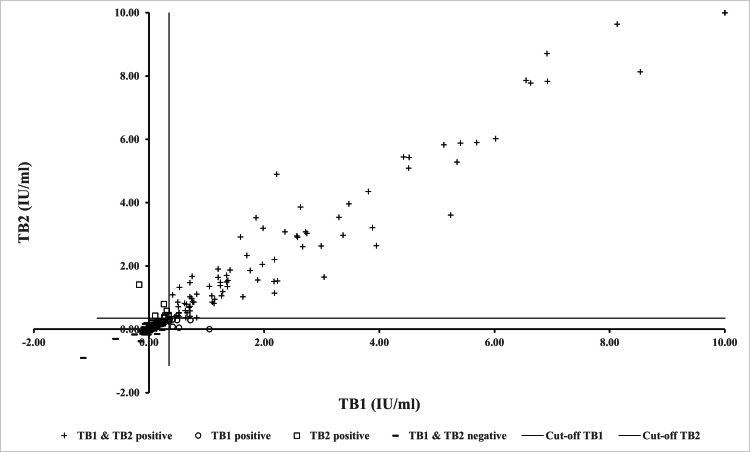
QFT-Plus results and the distribution of IFN-γ concentrations in TB1 and TB2 tubes. Scatterplot of IFN-γ concentrations in TB1 and TB2 tubes divided QFT-Plus results into concordantly positive (+); n = 93 (34.57%), discordant (○ for TB1 positive only; n = 8 (2.97%) and □ for TB2 positive only; n = 9 (3.35%)) and concordant negative (-); n = 159 (59.11%) groups. IU/ml: International unit per milliliter; Mdn: Median; n: Number; IFN-γ: Interferon-gamma.

Comparing the QFT-Plus results based on TB1 and TB2 detection, 93 (34.57%) were concordantly positive, and 159 (59.11%) were concordantly negative. Seventeen HCWs (6.32%) had discordant results, of which eight (2.97%) were TB1+/TB2-, and nine (3.35%) were TB1-/TB2+ discordant. The overall agreement between TB1 and TB2 detection results among HCWs was 252/269 (93.68%), with a Cohen’s κ coefficient of 0.87 (almost perfect; p-value < 0.05). The correlation between IFN-γ concentrations in TB1 and TB2 tubes was strong (Spearman’s ρ = 0.91; p-value < 0.05), reflecting the strong correlation between TB1 and TB2 detection. The discordant cases (TB1+/TB2- and TB1-/TB2+) all had median IFN-γ concentrations closer to the cut-off value (TB1+/TB2- cases: TB1 median = 0.45 (IQR 0.39-0.62) IU/ml, TB2 median = 0.27 (IQR 0.07-0.30) IU/ml; TB1-/TB2+ cases: TB1 median = 0.27 (IQR 0.26-0.31) IU/ml, TB2 median = 0.44 (IQR 0.42-0.58) IU/ml). The TB1/TB2 discordant results showed median IFN-γ levels lower than those of the concordant positive cases (TB1+/TB2+ cases: TB1 median = 1.76 (IQR 0.76-3.88) IU/ml, TB2 median = 1.70 (IQR 0.98-3.96) IU/ml) and higher than those of the concordant negative cases (TB1-/TB2- cases: TB1 median = 0.01 (IQR -0.01-0.08) IU/ml, TB2 median = 0.02 (IQR -0.01-0.10) IU/ml). A summary of QFT-Plus results and the distribution of IFN-γ concentrations in TB1 and TB2 tubes are presented in Figure 1.​​​​​​​

Factors associated with QFT-Plus positivity

The univariate ORs (crude odds ratios, OR) of being QFT-Plus positive or having LTBI for different characteristics are presented in Table 2. In the univariate analysis, HCWs aged > 40 years or having an employment duration > 10 years each showed a statistically significant association with QFT-Plus positivity or LTBI (OR = 3.21, 95% CI: 1.84-5.64, p-value < 0.05, and OR = 3.19, 95% CI: 1.66-6.37, p-value < 0.05, respectively). For HCWs in these categories, the odds of a positive QFT test were three times higher. These characteristics remained associated with a positive QFT-Plus result in the multivariate analysis (aOR = 2.05, 95% CI: 1.07-3.96, p-value < 0.05, and aOR = 2.34, 95% CI: 1.05-5.21, p-value < 0.05, respectively). Conversely, there was no significant association of QFT-Plus positivity or LTBI with other characteristics, which included gender (female, male), employment job (office clerk/housekeeper, registered nurse/nurse assistant, patient care personnel, or medical technologist), and working department (inpatient, outpatient, intensive care unit, or laboratory). Table 2 presents the crude OR and aOR for LTBI based on the uni- and multivariate analyses.

Factors associated with CD8+ cytotoxic T lymphocyte response

A difference in IFN-γ concentrations between the TB2 and TB1 tubes (TB2 - TB1) greater than 0.6 IU/ml was considered a true difference, which has been postulated as indicative of CD8+ cytotoxic T lymphocyte response and as a predictor for recent TB infection. We observed this true difference in 21 HCWs who tested positive for LTBI (n = 21/269, 7.81%). Univariate logistic regression analysis revealed that being male (OR = 3.93, 95% CI: 0.84-14.37) was the only predictor significantly associated with this true difference. However, no characteristics were significantly associated with both a QFT-Plus positive result and a true difference in IFN-γ concentrations between the TB2 and TB1 tubes greater than 0.6 IU/ml after the multivariate analysis, as shown in Table 3.

**Table 3:** Univariate and multivariate logistic regression analyses to identify factors associated with CD8+ T cell response. The difference in IFN-γ concentrations between TB2 and TB1 tube (TB2-TB1) >0.6 IU/ml was considered a true difference indicating the CD8+ T cell response. QFT-Plus: QuantiFERON-TB Gold Plus; IU/ml: International unit per milliliter; n: Number; OR: Odds ratio; aOR: Adjusted odds ratio. ^a^p-value <0.05 was considered statistically significant.

Characteristics	Total	TB2-TB1 > 0.6 IU/ml (n = 21)				
	(n=269)	n (%)	OR (95% CI)	p-value	aOR (95% CI)	p-value
Gender						
Female	251	17 (6.77)	Reference		Reference	
Male	18	4 (22.22)	3.93 (0.84-14.37)	0.02^a^	5.0 (0.91-27.54)	0.07
Age (years)						
≤ 40	114	5 (4.39)	Reference		Reference	
> 40	155	16 (10.32)	2.51 (0.84-9.01)	0.07	2.66 (0.89-8.0)	0.08
Employment						
Office clerk/Housekeeper	37	5 (13.51)	Reference		Reference	
Registered nurse/Nurse assistant	178	11 (6.18)	0.42 (0.12-1.66)	0.12	0.59 (0.18-1.92)	0.38
Patient care personnel	27	3 (11.11)	0.8 (0.11-4.6)	0.77	0.52 (0.09-3.08)	0.47
Medical technologist	27	2 (7.41)	0.51 (0.05-3.49)	0.44	0.18 (0.01-6.02)	0.34
Department						
Inpatient	115	6 (5.22)	Reference		Reference	
Outpatient	90	10 (11.11)	2.27 (0.71-7.9)	0.12	1.7 (0.55-5.28)	0.36
Intensive care unit	40	3 (7.5)	1.47 (0.23-7.3)	0.59	1.49 (0.35-6.36)	0.59
Laboratory	24	2 (8.33)	1.65 (0.15-10.02)	0.55	2.29 (0.07-77.11)	0.64
Employment duration (years)						
≤ 10	72	3 (4.17)	Reference		Reference	
> 10	197	18 (9.14)	2.31 (0.64-12.6)	0.18	ND	-

## Discussion

This study demonstrated the contribution of LTBI detection using QFT-Plus, revealing a high prevalence and risk factors for LTBI in this population. To date, QFT-Plus has been utilized only minimally in Thailand, with few Thai studies reporting its use for the diagnosis of LTBI. However, the diagnostic role of QFT-Plus for LTBI detection has been effectively demonstrated in this high-prevalence TB setting. In the present study, approximately 40% of HCWs tested positive for QFT-Plus and were categorized as having LTBI. This information serves as one of the few available baseline data sets on LTBI in Thai HCWs detected by QFT-Plus before the COVID-19 pandemic. As this study was conducted in a provincial hospital, the setting could represent most healthcare facilities in the country, where studied data are limited, and the contexts differ from those of university hospitals.

Given the high background prevalence of TB in the country, the risk of TB infection among Thai HCWs is estimated to be high, higher than that in the community because they encounter a larger number of TB patients during their work, often under suboptimal prevention conditions due to limited resources or working constraints. For HCWs who work in environments with close, frequent, and prolonged exposure to infectious TB cases, the risk of infection is particularly increased [14]. The acquisition of TB infection in HCWs might also occur outside the hospital setting, such as in the community or through household contact. Nevertheless, the majority of HCWs in TB-endemic settings regularly report significant work-related TB exposure. Considering the influence of the occupational environment on TB infection, it would be beneficial to include a group outside the hospital as a control sample. By using a case-control approach, the effects of occupational exposure and the rate of LTBI in non-hospital and general populations could be more accurately investigated. In this study, we were only able to enroll HCWs, both direct and non-direct healthcare providers, due to budget constraints and a focus on high-risk groups. However, we found an association between an increase in LTBI prevalence and the duration of employment, suggesting that positive results may be related to occupational TB exposure. Although identifying and treating active TB cases are key elements for TB control, this approach alone may not be sufficient to reduce TB infection and achieve TB elimination targets. Nevertheless, TB-infected HCWs may act as vectors, since LTBI may progress to active TB, leading to potential transmission of TB in the workplace and community. Therefore, diagnosis and appropriate management of LTBI, including treatment, should be considered and undertaken, especially in high-risk populations [15].

In various Thai settings, there are controversies regarding LTBI screening, suitable testing, and LTBI therapy. Over the past decade, screening for LTBI has not been undertaken even in high-risk populations like HCWs. The most recent and significant approach supporting LTBI management in Thailand towards achieving the End TB goal is the adoption of LTBI screening and treatment among high-risk populations in the national TB program, in accordance with WHO’s LTBI guidelines [5]. Enhancing the effectiveness and accessibility of diagnosis, including treatment of LTBI and TB infection control in the program, involves several components such as expanding LTBI screening, promoting new technologies for LTBI testing, encouraging LTBI treatment, promoting research, and strengthening infection control practices in healthcare facilities. A recent Thai study using QFT-Plus published in 2021 reported a 61.6% LTBI prevalence among Thai male prisoners [16], highlighting the high prevalence of LTBI in this setting.

Discrepancies in the prevalence of LTBI based on detection techniques have been reported. Both the TST and the IGRA are used in Thailand and other Southeast Asian countries, but the TST, a traditional test, is mostly performed due to its low cost and ease of use [17-18]. We used the QFT-Plus test, reported to be more specific for detecting LTBI. A recent systematic analysis indicated that LTBI prevalence ranged between 26.6% and 36.0% in high-TB incidence countries such as Sub-Saharan Africa, India, and Southeast Asia, with Southeast Asia having the highest LTBI prevalence [19]. These numbers are higher than the global prevalence reported at about 24.8% and 21% according to IGRA and TST results, respectively [19]. More recently, a 2024 meta-analysis reported that LTBI prevalence in Asian populations was about 21% and 36% according to IGRA and TST, respectively, with the Southeast Asian region still having the highest prevalence of LTBI based on IGRA [17]. This finding supports the importance of LTBI screening in this region and treating eligible individuals, while studies on LTBI in Thailand using IGRA, including QFT-Plus, are relatively scarce. Compared to previous studies in Thailand using different techniques, varied frequencies of LTBI in Thai HCWs were documented: TST at 69.17% [20], QFT-GIT at 19.05% [7], and T-SPOT.*TB* at 15.79% [21]. Indeed, LTBI frequency can be affected by the studied populations, timing, settings, and the different techniques used. It has been reported that TST caused more false-positive results compared to QFT testing due to the influence of BCG vaccination or other mycobacterial infections [7,22]. Recent studies reported the prevalence of LTBI among Thai HCWs and medical students assessed by QFT-Plus at 14.71% and 6.3%, respectively [23-24]. These figures were lower than the prevalence estimate of 32.73% in Thai nursing professionals working in a tertiary-care center reported recently [25] and lower than those in this study. The comparison also revealed variations in LTBI prevalence according to healthcare professionals. The variation in agreement between TST and QFT tests has also been reported [7]. Currently, the QFT-Plus test is available in Thailand with limited accessibility. The main challenges to the effective application of this test in low- and middle-income countries like Thailand may involve the laboratory infrastructure and personnel needed for their utilization, including the cost of tests. Using QFT-Plus or combining the TST for initial screening and then QFT for confirming TST positive results offers an alternative. The selection of an appropriate protocol for LTBI screening in each setting requires special consideration of cost, availability, practicality, and benefits.

Regarding studied samples, our data revealed that most participants were female (93.3%), relatively older with ages over 40 years (57.62%), and predominantly nurses or nurse assistants (66.17%), as they form the majority working in the hospital and agreed to participate. The findings showed a high prevalence of LTBI (> 40%) in nearly all groups except in younger (age < 40 years) and more recently employed HCWs (working duration < 10 years) (Table 2). Indeed, LTBI can occur upon contact with infectious TB patients with or without predisposing factors such as low immunity or individual susceptibility. These high estimates should call more attention to working conditions and raise concerns about potential exposures to TB and acquiring TB infection. Education regarding TB infection and prevention should also be increasingly provided. For HCWs, they should be aware of occupational risks for TB infection, and those with LTBI should pay attention to the current infection conditions. Although a majority of individuals with LTBI do not go on to develop active TB disease, it is important to regularly screen for TB and avoid predisposing factors that may increase the risks of TB progression.

Univariate and multivariate analyses were performed using logistic regression to assess the association between possible risk factors and QFT-Plus positive results. Several factors may increase the risks of acquiring TB infection, and the risks of LTBI differ among HCWs depending on local TB prevalence, healthcare facilities, and infection prevention and control measures [26]. Different risk factors for LTBI in HCWs were reported. A recent study in Peru using QFT-Plus reported that demographic factors associated with LTBI in HCWs included gender, time working in the hospital, and occupational exposure to TB [26]. In this study, essential factors such as sex, age of the HCWs, and their working duration were considered. Our multivariate analysis showed that only two variables, employment duration > 10 years and age > 40 years, were significantly associated with QFT-Plus positivity or LTBI (p-value < 0.05). Findings regarding sex as a risk factor for LTBI in HCWs have been inconsistent.

Considering the workplace, whether in an inpatient or outpatient facility, was not found to be associated with LTBI in this study, differing from a recent study reporting that working in an outpatient department was a risk factor for TB infection [25]. The outpatient department was previously reported to be at greater risk since it was at the forefront of the healthcare setting and highly exposed to TB patients each day [25,27]. The high risk probably involves the limited number of HCWs at the workplace in addition to their increased exposure to numerous TB patients. However, our results in this setting provided no evidence to support this impact on the associations. Meanwhile, screening for active or presumptive pulmonary TB patients in the outpatient areas followed by designing confined spaces or wearing masks appeared to be effective in reducing exposure to TB. Furthermore, strict exposure areas, rapid active case-finding, and early treatment of active TB could be important strategies to prevent or reduce TB infection among HCWs. Besides the working department, it should be noted that no significant relationship was found between the type of job and LTBI, contrary to previous studies reporting a significant risk for LTBI associated with working in critical hospital areas [28] or critical jobs that highly generate or directly expose to MTB aerosols [29]. We explain that most HCWs who work in high-risk procedure jobs are more likely to take stringent precautions to avoid infection, in contrast to those in low-risk jobs. In addition, acquiring TB infection depends on various determinants. The contact's closeness and the TB case's infectiousness were considered critical determinants for TB infection [26]. In HCWs working in critical-risk areas for TB infection or at high-risk jobs involving aerosol-contacting procedures, some might be in low-risk TB exposure due to good infection prevention practices. Other factors may be associated with LTBI, and there are several preventive components for TB infection control in the hospital. Risk assessment and management in high-risk populations are important for TB infection prevention to diminish TB. Overall, the variation in infection may involve the result of compliance with protective measures for infection control. Regarding employment duration, our finding on the impact of working time was similar to the previous study and systematic review reports on LTBI among HCWs which found the association of TB infection with the length of time working in healthcare facilities [13,26,30]. This effect can be explained by the longer working time increasing exposure to TB and, consequently, increasing the risk of acquiring TB infection. Given these risks, LTBI in HCWs should be focused, and occupational health control measures such as airborne infection control, regular LTBI screening and treatment, including risk management should be strengthened.

Laboratory analysis of IFN-γ detection demonstrated a high degree of agreement between TB1 and TB2 responses at 93.68% (n=252/269; κ=0.87; p-value < 0.05). Additionally, a high degree of correlation between IFN-γ concentrations in the TB1 and TB2 tubes was observed (Spearman’s ρ=0.91; p-value < 0.05) (Figure 1). Generally, the QFT-Plus TB1 tube is quite similar to the QFT-GIT TB antigen tube except that the TB1 tube does not contain the TB7.7 antigenic peptide [14]. Both tubes contain long ESAT-6 and CFP-10 peptides, primarily stimulating CD4+ helper TB cells, while the antigenic peptides in the TB2 tube induce the CD8+ T-cell response. This high concordance of detection results suggested that the QFT-Plus might be used in the same format as its predecessor, the QFT-GIT. Concerning discordance, the discordant cases showed median IFN-γ concentrations markedly closer to the cutoff value. According to the manufacturer, an additional tube (TB2) of the new QFT test could detect the CD8+ T cell which might indicate a recent TB infection suggesting an increased risk of developing active TB. The difference in IFN-γ production between the TB2 and TB1 tubes was determined in this study to estimate CD8+ T cell stimulation provided by the TB2 tube. Regarding the IFN-γ levels, the TB2 minus TB1 values > 0.6 IU/ml were considered to represent the true difference between the two MTB antigen tubes and implied a recent TB infection [9,10]. In this study, we found about 7.81% (n=21/269) of HCWs had true differences in IFN-γ levels in the TB1 and TB2 tubes. This result suggested a predicted recent infection, but we were unable to completely rule out the potential of a remote infection. It should be noted that our results did not show an association between the true differences and any characteristics that could be significant risk factors for CD8+ T cell response following the multivariate analysis. This analysis would be useful since HCWs with the highest risk of developing TB could be identified based on the true difference in IFN-γ levels between the two QFT-Plus antigen tubes, and those should be closely monitored for TB or given preventive therapy. The use of CD8+ T cell response in QFT-Plus detection for predicting a recent infection needs more studies. Another finding of our study was the determination of the need for LTBI screening among HCWs. We found a majority requesting LTBI screening preferably with the QFT test. However, the utility of this test in a high TB burden may be an issue because it is expensive and requires special equipment and skilled personnel. Only a small number of HCWs were able to participate in this study and undergo QFT-Plus testing due to the high cost of QFT-Plus and the limited budget.

There were some limitations to this study. Firstly, it was conducted in a single site and it was free of subject selection because the participants were recruited freely and voluntarily, so the results might not be applied generally to other settings with different characteristics of HCWs. Secondly, we could not assess the association between the QFT-Plus positivity and the given infection prevention procedures such as the use of PPE or N-95 mask or other potential risk factors because of the restriction as to a routine practice. This could potentially impact the identification of additional factors associated with LTBI. Furthermore, there were a few comparisons with other studies using the same diagnostic method with the same target populations due to the small number of similar studies in Thailand. Another limitation was no comparison of the QFT-Plus results with those of the conventional TST and no control subjects. Lastly, all participants had no previous baseline for LTBI screening, restricting the determination of prevalence trends, including conversion and reversion. On the other hand, this study was carried out to support TB control and provide data on LTBI screening in groups at high risk by using the QFT-Plus test kit in Thailand. The use of QFT-Plus to detect LTBI and the evaluation of associated factors for LTBI in a setting with a high TB burden were the primary strengths of this study. Future studies are required to address some other aspects of LTBI and QFT-Plus that have not been investigated in this study. Despite some limitations, our findings suggested that QFT-Plus was useful for LTBI screening including treatment guidance. The report should be able to encourage enhanced prevention measures and screening for LTBI in high-risk populations.

## Conclusions

The prevalence of LTBI in these HCWs was high according to QFT-Plus results, and the significant risk factors associated with LTBI were age over 40 years and employment duration over 10 years. Factors associated with the significantly increased CD8+ T cell response for predicting recent TB infection were not presented. It is important to screen HCWs in this setting for LTBI, particularly those with long employment durations and older ages. The high prevalence of LTBI suggests that LTBI management, such as regular screening and treatment, should be considered alongside the strengthening of preventive measures, especially in high-risk populations.
